# Infant Rule Learning: Advantage Language, or Advantage Speech?

**DOI:** 10.1371/journal.pone.0040517

**Published:** 2012-07-18

**Authors:** Hugh Rabagliati, Ann Senghas, Scott Johnson, Gary F. Marcus

**Affiliations:** 1 Department of Psychology, New York University, New York, New York, United States of America; 2 Department of Psychology, Harvard University, Cambridge, Massachusetts, United States of America; 3 Department of Psychology, Barnard College, New York, New York, United States of America; 4 Department of Psychology, University of California Los Angeles, Los Angeles, California, United States of America; University of Barcelona, Spain

## Abstract

Infants appear to learn abstract rule-like regularities (e.g., *la la da* follows an AAB pattern) more easily from speech than from a variety of other auditory and visual stimuli (Marcus et al., 2007). We test if that facilitation reflects a specialization to learn from speech alone, or from modality-independent communicative stimuli more generally, by measuring 7.5-month-old infants’ ability to learn abstract rules from sign language-like gestures. Whereas infants appear to easily learn many different rules from speech, we found that with sign-like stimuli, and under circumstances comparable to those of Marcus et al. (1999), hearing infants were able to learn an ABB rule, but not an AAB rule. This is consistent with results of studies that demonstrate lower levels of infant rule learning from a variety of other non-speech stimuli, and we discuss implications for accounts of speech-facilitation.

## Introduction

If the ability to acquire language is the product of evolution, what should it be like? The Darwinian notion of ‘descent with modification’ suggests that language is acquired through a mixture of mechanisms descended from other cognitive domains–which are thus domain general–and mechanisms that have been co-opted and modified for the specific domain of language acquisition [1], [2], [3]. Evidence for domain-general mechanisms is abundant. Children “fast map” both words and facts [4] and demonstrate categorical perception of both speech and non-speech sounds [5], [6]. “Statistical learning,” the extraction of transitional probabilities between sequential items, acts across a range of linguistic and nonlinguistic stimuli: 8-month-old infants can learn that ‘Ba’ precedes ‘Pa’ in a corpus of syllables [7], and may apply the same ability to tones and shapes [8], [9].

Language-specific mechanisms have proven more elusive. Marcus and colleagues [2], [10] have argued that infants’ ability to extract abstract rules and regularities from sequences–a *sine qua non* of language acquisition–might involve at least one learning mechanism that is specially tuned to language. In particular, 7-month-old infants appear to learn regularities more easily from speech than non-linguistic materials [10], [11]. But speech is not the only form language takes [12]. Here, we ask if the advantage for speech in rule learning reflects a mechanism tuned toward speech in particular, or for communication and language more broadly, by testing if infants are able to extract abstract rules from sign language-like gestures.

### Rule Learning in Infancy

To investigate infants’ ability to extract abstract rules, Marcus, Vijayan, Bandi Rao, and Vishton [13] familiarized 7-month-olds to sequences of syllables that followed a particular grammar (e.g. *ga ti ti, wo fe fe* for ABB). At test, infants listened longer to sequences that were inconsistent with the grammar (e.g., *la la ta*, which is AAB) than consistent sequences (e.g., *la ta ta*). Critically, the test syllables had not been used in training, suggesting that infants can extract an abstract rule, generalize it to novel stimuli, and discriminate it from other similar patterns.

Infants learn abstract rules from speech with alacrity. Seven-month-olds appear to extract and discriminate between ABB, AAB and ABA rules [13] and construct hybrid rules combining types and tokens (e.g., AdiA) [14]. Work using optical imaging indicates even newborns can detect ABB patterns [15]. But in contrast to their success learning transitional probabilities, 7-month-olds fail to learn rules from non-speech stimuli, including animal sounds, pure tones, notes of different timbre [10], and chords [11], suggesting that speech specifically facilitates learning.

That is not to say that rule learning is exclusive to speech. Dawson and Gerken [11] reported that 4-month-olds, but not 7-month-olds, can learn rules from chords. In addition, when triplets of dog pictures are presented simultaneously, 7-month-olds extract both ABB and ABA rules [16]. But while rule learning is certainly not all-or-none, the precise conditions under which infants learn regularities, particularly from *sequential* input, are yet to be established, and regularities in natural language are typically sequential.

Some rather broad generalizations are that infants extract rules more easily from certain stimuli than others, that certain rules are easier to extract than others, and that it is easier to discriminate between certain pairs of rules than others. Frank, Slemmer, Marcus and Johnson [17] showed that 5-month-olds learn rules that are jointly instantiated in shapes and syllables, but not rules from shapes alone. Johnson et al. [18] demonstrated that 8-month-olds can learn ABB rules from shapes, but not AAB or ABA, and that while 11-month-olds learn AAB, they fail to learn ABA (This difficulty is not likely due to encodability: Even 2-month-olds can learn transitional probabilities over the same shapes [8]). Finally, 8-month-olds provide evidence of learning an ABB rule from shapes when tested against ABA, but do not when ABB is tested against AAB, suggesting that they fail to incorporate serial order into rules extracted from non-linguistic stimuli. By contrast, infants learning rules from speech have no such difficulties with different rules, serial order, or discriminability.

In summary, while rule learning is clearly not exclusive to speech, the generalization that rule learning is at least preferentially evoked by speech appears to be valid, at least compared to the operation of statistical learning, which readily generalizes to sequences of tones [9] and shapes [8]. Why might infants privilege speech for rule learning?

One intriguing possibility is that the communicative aspect of speech might be critical. Abstract structural regularities are vital for human communication, and so infants may search for regularities in speech as part and parcel of an attempt to learn about what is being communicated. Previously tested stimuli, like tones or shapes, are typically not communicative. Under this account, rules should be readily acquired from any communicative signal, even non-auditory ones such as gesture and natural signed languages. Infants who can hear appear to be attuned to such signals. They can learn signed languages [19], [20], and perceive signed gestures in a comparable manner to speech (e.g., showing categorical perception [21]). If rule learning is specialized for communication, not just speech, infants should easily extract rules from this alternative modality.

Alternatively, speech may be privileged because infants are predisposed to attend to it [22], or because its familiarity facilitates the types of comparisons that are necessary to extract a rule. Saffran et al. [16] explained their results, where infants learn rule-bound patterns from familiar animals, in this latter way.

To test if the modality-independent communicative value of speech facilitates rule learning, we asked whether 7.5-month-old infants learn abstract rules from sign language-like gestures. These were constructed to be maximally analogous to the language-like syllables of Marcus et al. [13]. Neither set is fully reflective of a complete natural language, with proper syntax and semantics, but both cases reflect communication systems containing often-arbitrary tokens whose combinations are governed by regularities. Like speech, the gestures we used were human, distinct, and potentially communicative. But unlike speech they were novel to the infants viewing them. If infants preferentially analyze patterns in communicative stimuli independently of modality and familiarity, they should successfully extract rules here. But if speech itself is critical then rule learning should be more fragmentary, if it even occurs at all.

## Methods

### Participants

Twenty-four 7.5-month-old full-term infants participated (range: 214 days –243 days, *M* = 233 days, *SD* = 8.9). Infants had not been exposed to American Sign Language (ASL) at home, and were reported to have normal hearing abilities. All procedures were approved by New York University’s Committee on Activities Involving Human Subjects, and informed written consent was obtained from participants’ parents or guardians.

### Materials

We used analogous materials to Marcus et al. [10], [13], with synthesized spoken syllables replaced by color videos of a model performing sign language-like gestures. Each gesture consisted of a movement to a common place of articulation (bringing the hand up from the waist (off-screen) to the front of the face), with a fixed hand orientation. Throughout this movement, the model articulated one of twelve ASL handshapes, which was the only parameter to vary between tokens. Previous work has shown that hearing infants discriminate between different ASL handshapes [21].

Each token lasted approximately 1.33 s. From the start of the movement to the end of the articulation of the handshape took approximately 0.66 s, consistent with previous observations that manual gestures occurring at a rate of 1.5 Hz are treated as linguistic [23], [24]. Following articulation, the model brought her hand back down to her waist (0.66 s). The stimuli’s degree of naturalness compares with Marcus et al.’s (1999) synthesized speech stimuli. The model had studied ASL as an adult, but was not a native signer.

Tokens were separately recorded then combined into AAB or ABB sequences. To ensure natural-looking sequences, we recorded each token at least 50 times, and selected items matched for length of movement, place of articulation, body posture and head orientation. The model used a neutral but friendly facial expression. Tokens were edited and combined using Final Cut Pro (Apple Computer).

Four gestures were assigned as ‘A’ tokens, four as ‘B’ tokens, and then exhaustively combined into 16 AAB and 16 ABB training sequences. An additional two different ‘A’ and two different ‘B’ tokens were used for test trials. Supporting Information S1 contains a full list of gestures; Supporting Information S2 contains sample videos.

**Figure 1:**
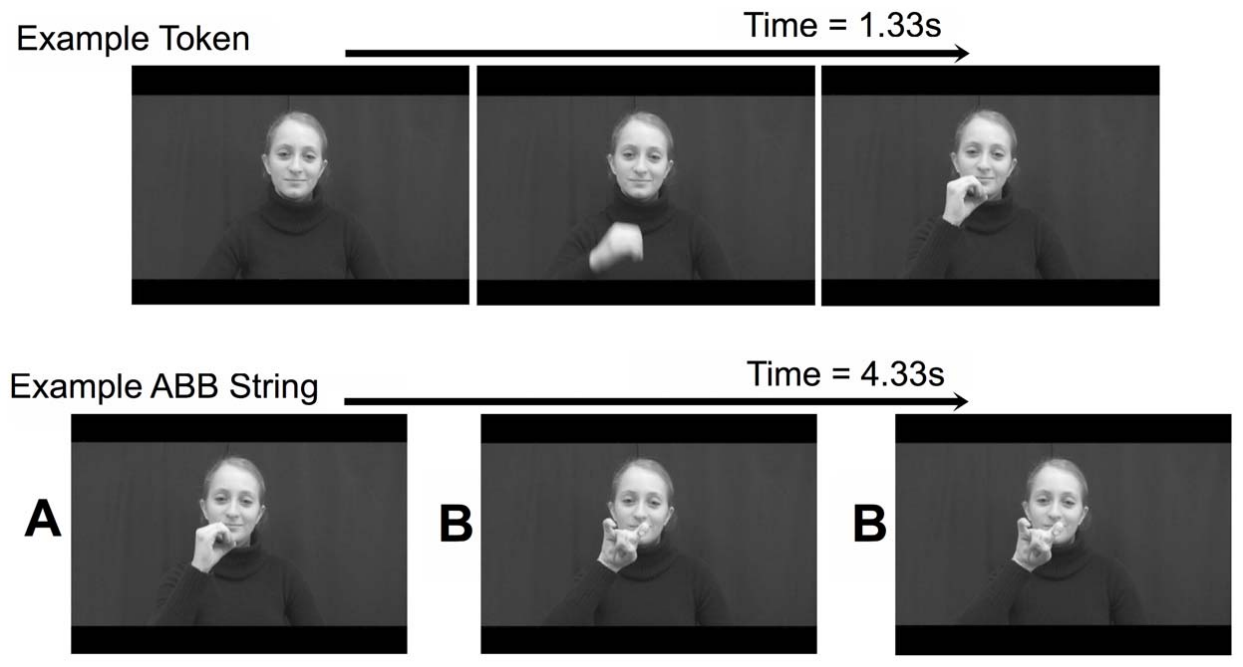
Example stills from a token and from an ABB sequence made up of three tokens.

Six freeze-frames (0.2 s) were added to the beginning of the first gesture of each sequence. Ten frames (0.33 s) of freeze-framed fade-out and 2 frames (0.07 s) of blackout were added to the end of each sequence. Sequences lasted 4.33 s. Figure 1 displays examples of a token and a sequence (actual stimuli were color movies).

### Procedure

Infants were tested on their parent’s lap, and familiarized to a training grammar (ABB or AAB) using a habituation procedure. Before each trial a beeping, moving blue and white target served as an attention-getter. Each training trial contained one of five videos of the 16 AAB or ABB training sequences presented in random order, constrained so that sequences could not be immediately repeated.

We used similar testing criteria as other work on visual rule learning [16], [18]: Training trials ended after a greater-than-2 s look-away or 120 s. Habituation was achieved when looking time across three consecutive trials (following the first three trials) summed to less than 50% of the looking time in the first three trials.

Eight test trials followed habituation, containing repetitions of one of two novel AAB or ABB test sequences. Order of presentation of the novel sequences and the consistent/inconsistent rule, as well as training grammar, were counterbalanced between infants.

## Results

If infants extract rules from any communicative stimuli, they should look longer towards sequences generated by a rule that is inconsistent with their training materials. Looking times were log transformed to reduce positive skew and heteroskedasticity, and analyzed using a 2*2 mixed analysis of variance, with training rule (AAB/ABB) as a between subject factor, and test trial type (consistent/inconsistent rule) as a within-subject factor. Mean number of trials to habituation was 8.9 (SD = 4.2), and mean looking time to habituation was 142.5 s (4.2).

**Figure 2:**
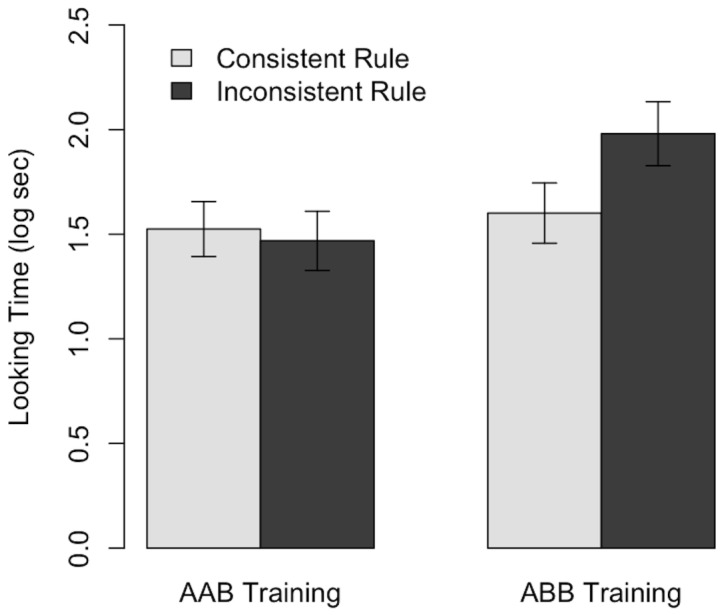
Looking times to consistent and inconsistent sequences during test trials, split by training rule.

Overall, infants did not look any longer to the inconsistent than the consistent test items (*F*(1,22)  = 2.56, *ns*), nor was there a reliable effect of training rule (*F*(1,22)  = 3.1, *ns*). However, there was a reliable test trial by training rule interaction, suggesting that infants were able to learn the ABB rule, but not AAB (*F*(1,22)  = 5.59, p = 0.027, see Figure 2).

Follow-up t-tests confirmed this. Infants trained on ABB looked reliably longer to the inconsistent items (M_consistent_ = 1.97 (0.56), M_inconsistent_ = 1.55 (0.49), *t*(11) = 2.57, *p* = 0.026), but infants trained on AAB exhibited no preference (M_consistent_ = 1.52 (0.46), M_inconsistent_ = 1.47 (0.49), *t*(11) = 0.51, *ns*).

Finally, to test whether a prior preference for AAB over ABB sequences might confound this result, we compared looking time during the entire habituation period. Infants did not look longer at AAB sequences; instead they looked longer at ABB sequences (*t*(22) = 2.7, *p* = .013), which is consistent with the premise that they were learning this rule. Still, this leaves an alternative explanation: Infants who looked longer during habituation learned the rule. To test this, we correlated mean looking time during habituation trials with the difference in looking time between the novel and familiar conditions. Habituation time did not reliably predict the size of this novelty preference (*r* = 0.32, *t*(22) = 1.6, *p = *.13); by contrast rule exposed to did (*r* = 0.45, *t*(22) = 2.4, *p = *.03).

In summary, given the opportunity to learn rules from communicative sign language-like gestures, infants’ performance was fragmentary. They could acquire an ABB rule but not AAB, a level of learning that clearly falls short of infants’ performance when learning rules from speech.

## Discussion

Our results suggest that the priority infants give to speech in rule learning does not extend to all potentially communicative signals. Whereas 7-month-old infants extract a variety of different rules from speech, 7.5-month-olds tested using sign language-like gestures apparently could extract an ABB rule and distinguish it from AAB, but could not do the reverse. This occurred even though the signs were natural human productions, discriminable, and clearly communicative, suggesting that rule-learning’s tuning for speech reflects some preference for the acoustic qualities of the signal. This tuning could be intrinsic or derive from experience with spoken language, but the present data indicate that it does not simply result from speech having a communicative quality.

Infants’ piecemeal rule learning here accords with previous reports on learning from non-linguistic stimuli [18]. Infants learning from shapes can extract an ABB rule and distinguish it from ABA, but when learning AAB they fail to generalize outside of their training space (in Marcus’s [25] terminology). This is not to say that infants learned nothing. It is likely that they recorded transitional probabilities between elements (statistical learning appears domain general). In addition, even if infants cannot extrapolate to new vocabularies, they might be able to interpolate within their familiar vocabulary (Marcus’s within-training-space generalization). While this possibility is often evaluated in the artificial grammar learning literature [26], [27], it has not been systematically explored in infant rule learning (though see [14], [28]). Clarifying exactly what infants learn is an important future task.

Johnson et al. [18] explained this ABB advantage in terms of working memory: A recency effect for sequence-final repetitions facilitates sequence-comparison. This can be contrasted with an attention-based hypothesis, in which ABB is easier because infants find sequence-initial changes (AB) more interesting and hence more learnable than repetitions (AA). Although the latter is logically possible, we favor Johnson’s explanation for three reasons. First, Johnson et al. demonstrated that 8-month-olds have no intrinsic preference for ABB or AAB without previous learning experience, counter to any notion of prior salience. Second, Endress, Dehaene-Lambertz and Mehler [29] have shown that repetitions can be more salient than transitions; for instance adults find it easier to learn rules based on repetitions than transitions. Finally, there are principled reasons for suspecting that repetitions are salient for infants; in particular the set of possible repetitions is much smaller (and hence rarer and more surprising) than the set of non-repetitions. Still, the two accounts are not mutually exclusive, and both possibilities remain open.

While the present pattern of piecemeal rule learning is qualitatively similar to Johnson et al. [18], infants also appeared to find it easier to learn rules from gestures than shapes: 7.5-month-olds distinguished ABB from AAB here, but 8-month-olds could not make the distinction for shapes. We concur with Saffran et al. [16] that familiarity with the stimuli might explain the difference, by highlighting abstract similarities between sequences that aid regularity learning. This requires that infants be more familiar with gestures than shapes, which seems a reasonable assumption.

Infants’ piecemeal nonlinguistic rule-learning abilities can therefore be explained if their developing memory and, possibly, attention systems are structured so that certain rules (e.g., AAB) are harder to learn, and if familiarity also serves to facilitate rule extraction (note that this account predicts that ABA rules, which should tax memory further still, should be even harder to learn). In summary, we think our data and account imply that there is no easy answer as to what makes a rule easy or hard to learn. Whatever the nature of the rule-learning mechanism, its operation is clearly constrained by a broad variety of factors.

While these factors may explain which rules are easier or harder to learn, they leave an open question as to what motivates infants to search for rules in the input. The hypothesis tested here was that infants probe for regularities in any communicative stimuli, but this was not supported by the data. Still, it is possible that infants might learn more readily in the presence of additional communicative cues that were absent in our materials. For instance, proceeding or concurrent verbalization might draw attention to the communicative nature of the stimuli. Alternately, different types of communicative gestures might permit rule learning. The gestures used here were (deliberately) unfamiliar to the infants, but it is possible that known gestures (points, waves, etc.) may be easier to process and thereby learn from. This is to say, we have by no means ruled out the existence of all communicative triggers.

An important remaining question is whether this gradient account can explain the speech-advantage at 7 months. Speech’s familiarity should lead to easier rule learning. However, recent work indicates that rule learning from speech is somehow specialized above-and-beyond the gradient account’s predictions. In particular, neonates identify an ABB rule in speech [15] but not comparable musical tones [30], suggesting that familiarity is not important for speech (although note that low-frequency acoustic information does penetrate the womb, providing some prenatal speech exposure).

In concert with our results, the studies above suggest that the speech advantage is neither the product of attention to a broad class of communicative stimuli, or some general improvement in “verbal” (or at least speech-specific) working memory. This leaves two accounts for future testing. Under one, speech is somehow special (e.g., [23]), and so infants are predisposed to learn from it. This predicts that a speech-bias should be universal across developmental contexts, including hearing infants growing up in households where signed languages are dominant. Moreover, the ability to learn rules from speech and from signs/gestures should also dissociate at younger ages.

Under the other account, hearing infants have rapidly learned that speech provides particular types of information that other stimuli do not (e.g., [11]), leading them to search for particular types of pattern–like rules–when listening to spoken language, and ignore that information for other stimuli. This latter account, but not the former, predicts that younger infants (say, at 3 months), even when not exposed to sign, should be able to learn rules from the sort of gestural stimuli used here. Moreover, any speech advantage will be particular to infants learning from speech, while sign will be special for infants learning from sign.

## Supporting Information

Supporting Information S1 List of handshapes used during gestures.
(DOC)

Supporting Information S2 Examples of gestures used in the training and test materials.
(ZIP)
