# Supplementary material for: Infant Rule Learning: Advantage Language, or Advantage Speech?
Source: PLoS One. 2012 Jul 18;7(7):e40517. doi: 10.1371/journal.pone.0040517 (PMC3399874; doi:10.1371/journal.pone.0040517)
Supplement: Supporting Information S1 — List of handshapes used during gestures. (DOC) [file pone.0040517.s001.doc]

**Supporting Information 1**

Gestures labeled following nomenclature in The American Sign Language handshape starter: A beginner's guide (Tennant and Brown, 2002).

*Training:* Bent-Five, U, 1-I, L, O, L-I, B, Six

*Test:* Five, V, H, I
